# Plants-Derived Biomolecules as Potent Antiviral Phytomedicines: New Insights on Ethnobotanical Evidences against Coronaviruses

**DOI:** 10.3390/plants9091244

**Published:** 2020-09-21

**Authors:** Arif Jamal Siddiqui, Corina Danciu, Syed Amir Ashraf, Afrasim Moin, Ritu Singh, Mousa Alreshidi, Mitesh Patel, Sadaf Jahan, Sanjeev Kumar, Mulfi I. M. Alkhinjar, Riadh Badraoui, Mejdi Snoussi, Mohd Adnan

**Affiliations:** 1 Department of Biology, College of Science, University of Hail, Hail PO Box 2440, Saudi Arabia; mousa.algladi@gmail.com (M.A.); badraouir@yahoo.fr (R.B.); snmejdi@yahoo.fr (M.S.); drmohdadnan@gmail.com (M.A.); 2 Department of Pharmacognosy, Faculty of Pharmacy, “Victor Babes” University of Medicine and Pharmacy, 2 Eftimie Murgu Square, 300041 Timisoara, Romania; 3 Department of Clinical Nutrition, College of Applied Medical Sciences, University of Hail, Hail PO Box 2440, Saudi Arabia; amirashrafy2007@gmail.com; 4 Department of Pharmaceutics, College of Pharmacy, University of Hail, Hail PO Box 2440, Saudi Arabia; afrasimmoin@yahoo.co.in; 5 Department of Environmental Sciences, School of Earth Sciences, Central University of Rajasthan, Ajmer, Rajasthan 305817, India; ritu2735@gmail.com; 6 Bapalal Vaidya Botanical Research Centre, Department of Biosciences, Veer Narmad South Gujarat University, Surat, Gujarat 395007, India; patelmeet15@gmail.com; 7 Department of Medical Laboratory, College of Applied Medical Sciences, Majmaah University, Al Majma’ah 15341, Saudi Arabia; jahan149@gmail.com; 8 Department of Environmental Sciences, Central University of Jharkhand, Ranchi 835205, India; sanjivk845@gmail.com; 9 Saudi Center for Disease Prevention and Control, Al Aarid, King Abdulaziz Rd, Riyadh 13354, Saudi Arabia; drmulfi@gmail.com; 10 Section of Histology-Cytology, Medicine College of Tunis, University of Tunis El Manar, La Rabta-Tunis 1007, Tunisia; 11 Laboratory of Histo-Embryology and Cytogenetic, Medicine College of Sfax, University of Sfax, Sfax 3029, Tunisia; 12 Laboratory of Genetics, Biodiversity and Valorization of Bio-Resources, Higher Institute of Biotechnology of Monastir, University of Monastir, Monastir 5000, Tunisia

**Keywords:** SARS-CoV, COVID-19, coronavirus, medicinal plant, phytomedicine, ethnobotany, antiviral, natural products, bioactive compounds

## Abstract

SARS-CoV-2 infection (COVID-19) is in focus over all known human diseases, because it is destroying the world economy and social life, with increased mortality rate each day. To date, there is no specific medicine or vaccine available against this pandemic disease. However, the presence of medicinal plants and their bioactive molecules with antiviral properties might also be a successful strategy in order to develop therapeutic agents against SARS-CoV-2 infection. Thus, this review will summarize the available literature and other information/data sources related to antiviral medicinal plants, with possible ethnobotanical evidence in correlation with coronaviruses. The identification of novel antiviral compounds is of critical significance, and medicinal plant based natural compounds are a good source for such discoveries. In depth search and analysis revealed several medicinal plants with excellent efficacy against SARS-CoV-1 and MERS-CoV, which are well-known to act on ACE-2 receptor, 3CLpro and other viral protein targets. In this review, we have consolidated the data of several medicinal plants and their natural bioactive metabolites, which have promising antiviral activities against coronaviruses with detailed modes of action/mechanism. It is concluded that this review will be useful for researchers worldwide and highly recommended for the development of naturally safe and effective therapeutic drugs/agents against SARS-CoV-2 infection, which might be used in therapeutic protocols alone or in combination with chemically synthetized drugs.

## 1. Introduction

Coronaviruses (CoVs) are single-stranded RNA viruses under the family of *Coronaviridae*; first reported in 1960 [1]. CoVs can infect humans and a wide range of animals including camels, cattle, cats, and bats, causing respiratory, hepatic, neurological and gastrointestinal diseases [2,3]. CoVs are further divided into subfamily *Orthocoronavirinae,* and classified into four genera; alpha, beta, gamma and delta-CoVs [4,5]. However, to date, only seven types of human-CoVs (HCoVs) exist that can infect humans. They are Middle East Respiratory Syndrome-CoV (MERS-CoV), Severe Acute Respiratory Syndrome-CoV (SARS-CoV), HCoV-229E, HCoV-OC43, HCoV-NL63, HCoV-HKU1, and novel SARS-CoV-2 [4,5]. In early December 2019, novel SARS-CoV-2 first appeared in the city of Wuhan, China with symptoms of severe viral pneumonia [6,7]. The disease, caused by the novel SARS-CoV-2, has been given the name COVID-19 by the World Health Organization (WHO) [8]. In a short period of time, WHO issued guidelines for patients monitoring, sample collection, and various other detailed pieces of information on this pandemic disease [8]. Many reports confirmed the spread of SARS-CoV-2 by human to human transmission [5,6,7]. However, there are no specific drugs or vaccines currently available to cure COVID-19 infection. Thus, this scenario reflects the urgency and need to develop new drugs or vaccines against COVID-19 infection [9].

**Figure 1:**
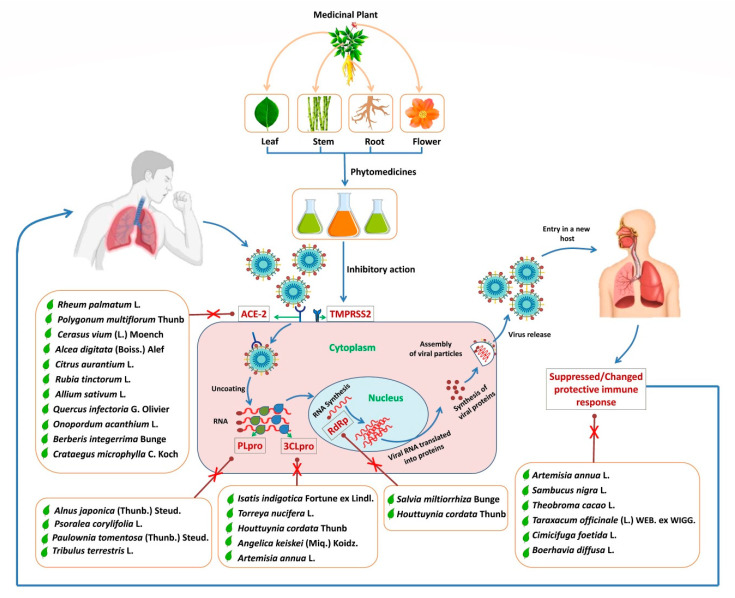
Diagrammatic representation of various medicinal plants and their possible specific inhibitory sites to act against SARS-CoV-2.

Under the current, difficult context, almost 27,236,916 people have been infected by SARS-CoV-2, and approximately 891,031 deaths has been reported as of 7 September 2020 due to COVID-19 infection globally [8,10,11]. The question raised here is “why researchers and global scientific community have also not taken in consideration the treatment of SARS-CoV-2 infection through natural plant-based compounds or usage of ethnomedicinal medicinal plants?”. Though we cannot bring about an instant cure, considering the urgency of eradicating the COVID-19 pandemic, this paper will open new perspectives about plant molecules in developing various strategies against infectious diseases [12,13]. Mother Nature has always been considered as the prime source for the discovery of unique and new bioactive compounds, which have helped in combatting various diseases and infections [14,15,16,17,18,19,20,21,22]. Medicinal plants are known to produce innumerable bioactive metabolites with different pharmacological properties, including anticancer, anti-inflammatory, antimicrobial, antioxidant, anti-malarial, anti-hypertension, anti-ageing, anti-diabetic, anti-hyperlipidemia, anti-osteoporotic, hepato-protective, immunomodulator, etc. [23,24,25,26,27,28]. Many are known to have proven antiviral effects with the ability to inhibit viral replication, and can cure various types of viral infections [13,29,30,31]. In this review, we have focused on specific medicinal plants and their bioactive compounds, which are known to use/can be used against coronaviruses, HIV, and other viruses to prevent the replication of viruses and reduce the viral load. In addition, how these medicinal plants and their derived biomolecules will be beneficial for the management of SARS-CoV-2 infection is also discussed; a repository of possible and effective antiviral plant candidates has also been made (Figure 1).

## 2. Therapeutic Potential of Medicinal Plants against SARS-CoV and MERS-CoV Infections

There are various medicinal plants which are known to have an inhibitory effect against SARS-CoV, HCoV-22E9, MERS-CoV and other viral infections (Table 1). They were chosen specifically due to their mode of action and potency, and have been used and researched with ethnobotanical evidence against coronaviruses or other viruses (HIV, Influenza, etc.). Coronaviruses belong to positive sense RNA viruses and mostly use the ACE-2 (Angiotensin-converting enzyme-2) receptor, 3CLpro (3 Chymotrypsin-like protease), PLpro (Papain-like protease), RdRp (RNA-dependent RNA polymerase) enzyme and other known factors to gain entry into the human cell and complete the life cycle. Thereby, all these selected plants have been tested by various researchers globally to act on these specific target proteins and receptors, and, moreover, inhibit RNA replication in the other viruses too. This was the chief rationale in selecting these plants, which are described in detail below with their mode of action, which may also possibly be considered as a therapeutic choice against SARS-CoV-2. This presentation is designed in order to open new pathways towards the management of highly contagious diseases with the help of natural compounds.

### 2.1. Bupleurum Species

*Bupleurum* plant species are extensively dispersed in the northern hemisphere and are used as one of the oldest phytomedicines in China. Many reports have identified the activity of this herbal plant in the treatment of HCoV-22E9 and other viral infections [32,33]. Generally, *Radix bupleuri* (*R. bupleuri*) is derived from the dried roots of *Bupleurum* species and used for the treatment of various diseases [34]. It has great pharmacologically significant activities, the main ones reported in the literature being: antiviral, anti-inflammatory, anti-tumor, neuro-modulation and immunoregulation [34,35]. Approximately 7% of naturally occurring saikosaponins (triterpene saponin glycosides) are present in *R. bupleuri,* which is the main component of this medicinal plant with potent effects. Four types of saikosaponins (SS) are found; SSa, SSb2, SSc and SSd, which are responsible for the most pharmacological activities in this medicinal plant [36]. The SSa, SSb2 and SSd have potential to inhibit the effects against coronavirus 229E, SARS-CoV and influenza A virus [35]. Moreover, the mechanism of action of these SS employing antiviral activity interrupts the early stage of viral replication inside the host cells [35]. In addition, these SS also attenuate pro-inflammatory cytokines production, inhibiting viral replication through down-regulating NF-kB signaling, caspase 3-dependent virus ribonucleoprotein nuclear export, lung neutrophil and monocytes recruitment in an experimental in vivo mice model [35].

### 2.2. Lycoris radiate *(L’Hér.) Herb.*

*Lycoris radiata* (*L. radiata*) belongs to the *Amaryllidaceae* family and originally it was found in China, Korea, Japan and Nepal [37]. This medicinal plant has wide-ranging biological activities comprising: antiviral, anticancer [37], anti-malarial [38], anti-inflammatory [39] and induction of nausea and emesis [40]. Additionally, and most importantly, *L. radiata* has been known to have antiviral effects on SARS-CoV [41,42], poliovirus, human immunodeficiency virus (HIV), measles virus, herpes simplex virus and coxsackie virus [42,43]. Its potent bioactive compound is lycorine, which is extracted from the flower and stem cortex of *L. radiata* plants. Currently, this plant is in use for the treatment of various diseases due to its broad-spectrum biological activities. It has also been recommended as a promising medicinal plant for the development of potential drugs against SARS-CoV infection [42,43]. The antiviral mechanism of action of this plant is by inhibiting virus replication in the cells through inhibiting autophagy [43]. Moreover, JNK/MAPK signaling pathway is closely connected to autophagy, and through this signaling pathway, the plant extract inhibits the process of autophagy due to reduced JNK phosphorylation induced by viral replication [43,44].

### 2.3. Artemisia annua *L.*

This Chinese medicinal plant has been used for a long time to treat various diseases such as bronchitis and hemorrhoids, and is potentially effective for its anti-malarial, antiviral, anticancer, etc., properties [45,46,47]. However, *Artemisia annua* (*A. annua*) has been known to possess antiviral activity and currently is in use for the treatment of Poliovirus, HIV, RSV, HSV1, hepatitis C, type 2 dengue virus and human cytomegalovirus [29,48]. *A. annua* contains quercetine, flavonoid, polyphenols, triterpenes, sterols, saponins, polysaccharides, dicaffeoylquinic acid and other molecules [48]. Due to the presence of these molecules, *A. annua* extracts (whole plant) have shown an important role, being assigned with immunomodulator, antiviral, antioxidant and anti-inflammatory properties. Moreover, these compounds/molecules have been known to inhibit the enzyme activity of 3CLPro [49,50]. Previously, this medicinal plant has been used to treat SARS-CoV and MERS infections [50], and is currently being used against novel SARS-CoV-2 infection [49]. The mechanism of action of *A. annua* is to inhibit the enzymatic activity of 3CLPro, which is also produced by SARS-CoV-2, and increase the production of pro-inflammatory cytokines prostaglandin E2 (PGE2), IL-6, TNF-a, IFN-γ and enhance the genesis of CD4^+^ and CD8^+^ T cell populations [49,50,51].

### 2.4. Pyrrosia lingua *(Thunb.) Farw.*

*Pyrrosia lingua* (*P. lingua*) belongs to the *Polypodiaceae* family and mostly occurs in China, Japan, Korea and other Asian regions [52]. *P. lingua* is known for its antiviral, antioxidant, antibacterial and anticancer activities; it even stops the formation of urinary calculi [52,53]. Furthermore, it contains several bioactive components, such as flavonoids, chlorogenic acid, mangiferin, isomangiferin, astragalin and trifolin [54]. The extract of *P. lingua* leaves has been used by many researchers for the treatment of HIV, SARS and other viral infections [50,52,53]. In the case of SARS-CoV-1, this plant has shown the ability to inhibit viral infection, but the mechanism of action is still not clear [48,50].

### 2.5. Isatis indigotica *Fortune ex Lindl.*

*Isatis indigotica* (*I. indigotica*) is a very old Chinese herbal plant belonging to the *Cruciferae* family. It is mostly found in China, Hong Kong, Taiwan and other regions of Asia [55]. According to Lin et al., *I. indigotica* has the potential to inhibit/block SARS-CoV-1 entry and replication in its host [48]. However, the research group used *Radix isatidis* (dried root) of *I. indigotica* for extracting potent compounds for the treatment of SARS-CoV-1-infected patients. Furthermore, its root contains indirubin, indican, indigo, sinigrin, β-sitosterol, hesperetin, aloe-emodin and many more bioactive compounds [55,56]. According to one in vitro study, all these extracted compounds were used against SARS-CoV-1 infection, and it was found that indigo, sinigrin, aloe-emodin and hesperetin were able to inhibit the virus entry and replication by inhibiting the SARS-CoV-1 3CLpro [57]. We know that coronavirus 3CLpro mediates the proteolytic processing of replicase polypeptides into the functional proteins and plays a key role in viral replication [58]. Therefore, *I. indigotica* can also be considered as a potential therapeutic choice against SARS-CoV-2.

### 2.6. Torreya nucifera *L.*

This plant is mostly found in snowy areas near the Sea of Jeju Island in Korea, and is considered as a traditional medicinal plant. Its leaves are mostly used for the treatment of stomachache, hemorrhoids and rheumatoid arthritis [59,60]. During SARS-CoV-1 infection, Young Bae Ryu et al. used *Torreya nucifera* (*T. nucifera*) plant leaves for in vitro experiments, and the results showed a potential inhibitory effect [48,61]. Ryu et al. isolated 12 phytochemical compounds from the ethanol extract of the *T. nucifera* leaves. Only the biflavonoid amentoflavone showed efficacy against SARS-CoV-1 [61]. This biflavonoid of *T. nucifera* has the potential to block the activity of 3CLpro of the coronavirus and can inhibit the viral replication [61].

### 2.7. Houttuynia cordata *Thunb.*

This Southeast Asian plant belongs to the family of *Saururaceae*, which is traditionally used for the treatment of lung disorders such as cough, lung abscess, phlegm, and dyspnea. *Houttuynia cordata* Thorn (HCT) is a Chinese herbal plant well-known for its potent effects in the treatment of pneumonia, refractory hemoptysis, and SARS-CoV-1 infection [62,63]. It has anti-inflammatory, anti-allergic, antioxidant and anticancer properties [62]. The bioactive compounds present in HCT are comprised of rutin, hyperin, isoquercitrin, quercetin, afzelin, reyoutrin, kalium sulfuricum, cordarine, decanoyl acetaldehyde, lauric aldehyde, myrcene, α-pinene, methyl nonyl ketone, d-limonene, linoleic acid, aspartic acid, palmitic acid, water-soluble polysaccharides, amino acids, vitamins, manganese, potassium, zinc, iron and copper [64,65,66]. During the SARS-CoV-1 infection, the leaves of this Chinese medicinal plant were used to treat patients and showed good efficacy against SARS-CoV-1 [48,57]. Lau et al. conducted one experiment using HCT against SARS-CoV-1. Results showed that HCT can inhibit SARS-CoV-1 activity including an immunomodulatory effect [63]. However, the mode of action of HCT is to inhibit the 3CLpro activity of SARS-CoV-1 and obstruct the activity of RdRp [63]. Hence, it can block the entry of the virus and impede viral replication [63]. This inhibitory mechanism makes HCT a good choice to be used against SARS-CoV-2 infections.

### 2.8. Lindera aggregate *(Sims) Kosterm.*

*Lindera aggregate* (*L. aggregate*) is a traditional Chinese medicinal plant belonging to the *Lauraceae* family and mostly found in China and Japan [67]. The root of this plant is mostly used to treat chest pain, inflammation, indigestion, cold hernia and other diseases. It contains several bioactive components, such as flavonoids, isoquinoline alkaloids, sesquiterpene lactones and tannins [68,69]. Moreover, *L. aggregate* has also showed other biological activities such as antiviral, anti-tumor, anti-inflammatory, antimicrobial and anti-diabetic activities [48,69,70]. *L. aggregate* leaves can also be used to drink as tea, due to their protective effect against oxidative stress [70]. In 2005, Shi-you Li et al. investigated the effect and efficacy of *L. aggregate* roots against SARS-CoV-1 [50]. An in vitro study showed that *L. aggregate* is able to inhibit SARS-CoV-1 with EC_50_ value of 88.2 ± 7.7 µg/mL [50]. However, the mode of action is still not clear, but it was suggested that *L. aggregate* roots can possibly inhibit the viral replication and block the entry of virus [50].

## 3. Known Medicinal Plants Acting on ACE-2 Receptor

As of recently, it is known that SARS-CoV-2 is using the ACE-2 receptor to enter into human cells. There are various medicinal plants which have the potential to act on the ACE-2 receptor and are well-known for blocking the transmission or entry of CoVs. After an in-depth literature search, several plants have been found to act on the ACE-2 receptor, which could become promising antiviral agents and can help in combatting COVID-19 pandemic. They are Radix et Rhizoma Rhei, *Radix Polygoni multiflori*, *Caulis Polygoni multiflori*, *Cerasus avium* (L.) Moench, *Alcea digitata* (Boiss.) Alef, *Rubia tinctorum* L., *Citrus aurantium* L., *Berberis integerrima* Bge, *Peganum harmala* L. and *Allium sativum* L. [78,91].

### 3.1. Rheum palmatum *L.*

This herbal plant belongs to the family *of Polygonaceae*. It is mostly found in mountainous regions with high elevations, such as the Sichuan, Gansu and Shaanxi regions of China [81]. It is effectively used as a laxative or astringent for the treatment of stomachache, hemorrhoids, liver bile disease or gastroenteritis [243]. It contains some potent bioactive compounds including emodin, physcion, chrysophanol, rhein and aloe-emodin [80,82]. Known biological activities are antiviral, anti-pyretic, anti-neoplastic, anti-spasmolytic, antibacterial, laxative, hemostatic and anti-spasmodic [79,81,82,244]. It was also used against SARS-CoV-1 infection, due to its potential efficacy for acting on the ACE-2 receptor, leading to blockage of viral entry into cells and replication of the CoVs [78,80]. An in vitro study conducted by Ho et al. 2007 showed the potential of Radix et Rhizoma Rhei (root tubers of *Rheum palmatum* L.) in blocking the entry of SARS-CoV-1 to inhibition sites such as the ACE-2 receptor [78]. Furthermore, the major active component of this plant is emodin, which is responsible for blocking the binding of SARS-CoV-1 S protein to ACE-2 receptor [78,80]. Therefore, the use of emodin extracted from Radix et Rhizoma Rhei can be considered for the possible therapeutic management of COVID-19. This will possibly provide us with new insight into therapy against SARS-CoV-2.

### 3.2. Polygonum multiflorum *Thunb*

*Polygonum mulitflorum* Thunb (PMT) is mostly found in China, Korea and Japan, belonging to *Polygonaceae* family [245]. Radix Polygoni multiflori (root tubers of PMT) is mostly used in treating many kinds of diseases, such as rubella, scrofula, waist and knee pain, paralysis, vaginal discharge, hypercholesterolemia (liver and kidney), malaria, and various other diseases, possessing neuro-protective, antioxidation, immunomodulation, anti-hyperlipidemia, anticancer, heap-toprotection, anti-inflammation, and anti-CoV functions [86,87,246]. The potent bioactive compounds present in PMT which are responsible for the therapeutic effects against various diseases are listed in Table 1 [84,85,88,247]. However, Ho et al. found that emodin is the most effective compound against SARS-CoV-1. The data were published to show the potential and efficacy of PMT in blocking the entry of SARS-CoV-1 by acting on the ACE-2 receptor [78]. The mode of action of PMT is similar to *Rheum palmatum* L and the major active constituent is found to be emodin in both plants. Therefore, it is highly recommended to focus on emodin for possible and effective management of SARS-CoV-2 infection, combination with other therapeutic approaches.

### 3.3. Cerasus avium *(L.) Moench*

This Persian medicinal plant belongs to the *Rosaceae* family and is mostly used as an antioxidant, antimicrobial and antiviral [93]. Its stem contains polyphenols, carotenoids, vitamins, minerals and many other bioactive components [90,248]. However, this plant has strong potential to act on the ACE-2 receptor and block the further processing of the viruses [78,92,94]. According to Ziai et al.’s 2009 in vitro study, this plant showed very good potential to inhibit or completely block the ACE-2 receptor [94]. Subsequently, Heidary et al., 2020 recently suggested that this plant has good potency against SARS-CoV-2 and must be used for the treatment of its infection [91].

### 3.4. Alcea digitata *(Boiss.) Alef*

*Alcea digitata* (*A. digitate*) is a Persian medicinal plant belonging to the *Malvaceae* family with antiviral, antioxidant, anti-inflammatory, antimicrobial, anti-tussive, expectorant and laxative therapeutic effects [96,97]. The flowers of *A. digitata* have been used for lung and respiratory disorders, head and neck cancer, and lubrication of the throat [97]. According to one published report [96], *A. digitata* is known to have good potential to block or inhibit the ACE-2 receptor. Recently, Heidary et al., 2020 suggested that *A. digitata* can possibly show good inhibitory effects against SARS-CoV-2 infection [91].

### 3.5. Citrus aurantium *L.*

*Citrus aurantium* (*C. aurantium*) belongs to the family of *Rutaceae* and is generally known as bitter orange [249]. This plant is known to have many essential components with biological effects [100,101], such as phenolics (flavanone glycosides, hydroxycinnamic acids), vitamin C, and carotenoids [99,249,250]. However, *C. aurantium* fruit extract is manly used for the treatment of anxiety, lung related diseases, obesity, gastrointestinal disorders and prostate cancer [249,251], buts has potential to inhibit or block the ACE-2 receptor. Some in vitro studies have shown its efficacy in inhibiting ACE-2 receptors [91].

### 3.6. Rubia tinctorum *L.*

*Rubia tinctorum* (*R. tinctorum*) is mostly found in Southern Europe, Western Asia and North Africa and belongs to the family of *Rubiaceae* [252]. *R. tinctorum* is mostly used to treat kidney and bladder stones, and menstrual and urinary disorders [104,253]. Furthermore, the root of *R. tinctorum* contains red color due to the presence of anthraquinone, alizarin and pseudopurpurin, which is also used for dyeing purposes [103]. On the other hand, *R. tinctorum* has shown potential to inhibit or block the ACE-2 receptor [91]. in vitro studies revealed the efficient use of *R. tinctorum* to inhibit ACE-2 receptors [91].

### 3.7. Allium sativum *L.*

The common name of *Allium sativum* (*A. sativum*) is garlic, and it belongs to the *Amaryllidaceae* family. *A. sativum* use for human welfare has been reported for thousands of years in the form of a spice [108]. It is an aromatic herbaceous plant and is consumed worldwide as a food as well as a remedy for different diseases [108]. *A. sativum* is reported to have numerous biological properties, such as antibacterial, antifungal, anti-carcinogenic, antioxidant, anti-diabetic, reno-protective, anti-atherosclerotic, and anti-hypertensive effects. Cloves of this traditional medicinal plant contain several potent components, such as alliin, allicin, ajoenes, vinyldithiins, and flavonoids [108,109,254,255,256], due to which it is mostly used for treatment of various disorders [106,107,108,109,257]. On the other hand, an in vitro study conducted by Ziai et al., 2009 on *A. sativum* and its potential efficacy to inhibit the ACE-2 receptor reported some effective results [94].

### 3.8. Quercus infectoria *G. Olivier*

*Quercus infectoria (Q. infectoria)* is commonly known as gall oak and belongs to the family of *Fagaceae* [112]. This medicinal plant is traditionally used for the treatment of diarrhea, menorrhagia, dysentery, gonorrhea, tonsillitis, impetigo and internal hemorrhages [112,258]. Bioactive constituents of *Q. infectoria* gall extract include phenolic compounds (catechol, *p*-hydroxybenzoic acid, caffeine, catechin, pyrogallol, e-vanillic acid, 3-hydroxytyrosol cinnamic, *p*-coumaric, gallic acids and resveratrol), flavonoids (naringin, rutin, 7-hydrohyflavone and hispertin) [111,259,260] with biological activities such as antiviral, antifungal, antibacterial, antioxidant, anti-inflammatory, anti-diabetic, anti-parasitic, anti-venom, etc. [112,114,261]. *Q. infectoria* has also shown strong potential to completely block the ACE-2 receptors due to the presence of many potent and tannin active components in vitro [113]. Similarly, this medicinal plant can also be considered for combinational therapeutic approaches in controlling the COVID-19 pandemic directly or indirectly.

### 3.9. Onopordum acanthium *L.*

*Onopordum acanthium (O. acanthium)* basically belongs to a family of *Asteraceae* and is commonly known as Scotch thistle [118]. It is found all over the world [262]. The biological activities of *O. acanthium* include antiviral, anti-tumor, anti-inflammatory and antioxidant effects. Extracts from the leaf, flower, stem and root of *O. acanthium* are also used as cardiotonic agents. *O. acanthium* contains many bioactive components, such as flavonoids, triterpenoids, lignans, phenylpropanoids, sesquiterpene lactones, and sterols [116,117,262]. Moreover, *O. acanthium* has shown efficacy to completely inhibit the activity of ACE-2 due to the presence of tannin bioactive components, as demonstrated by Sharifi et al., 2013 in his in vitro study. This makes it a considerable choice to test against SARS-CoV-2.

### 3.10. Berberis integerrima *Bunge*

*Berberis integerrima* (*B. integerrima*) belongs to the family of *Berberidaceae,* with different parts of the plant showing different colors [121]. It is mostly found in Iran and contains many types of alkaloids [122]. Bioactive components extracted from the root of *B. integerrima* include berbamine, berberuin, palmatine, oxyacanthine, malic acid, ascorbic acid, caffeic acid, ursolic acid, coumarin, beta-carotene and tannin [120,122]. *B. integerrima* possesses many bioactive properties, such as antiviral, anti-inflammatory, anti-hyperglycemic, anti-hyperlipidemic, anticancer, and antioxidant effects, as well as being a liver protective agent [120,121,122]. Moreover, this medicinal plant was tested by Sharifi et al., 2013 in vitro. His team showed that the usage of a 330 µg/mL concentration of *B. integerrima* was able to inhibit the ACE-2 receptor due to 88.2 ± 1.7 IC_50_ [113]. Therefore, *B. integerrima* can be further investigated for its potent medicinal values and may provide fruitful results against SARS-CoV-2.

### 3.11. Crataegus microphylla *C. Koch*

This medicinal plant belongs to the family of *Rosaceae* and almost all parts of the plant are used for remedial purposes [126]. It is widely used for the treatment of many diseases, including heart muscle cells activation, coronary dilation, regulated blood flow, use as an antioxidant and anti-diabetic, and many others [125,126]. It contains flavonoids (phenols, phenolic acids, procyanidins, flavonoids, triterpenes, polysaccharides, catecho-lamines) which help in controlling/regulating various diseases [120,124,125,126]. Furthermore, it has also showed efficacy to inhibit the ACE-2 receptor and prohibit the entry of virus into the cell [113]. in vitro results suggested the use of a 330 µg/mL concentration of *Crataegus microphylla* was able to inhibit the virus binding to the ACE-2 receptor, and their IC_50_ was observed as 80.9 ± 1.3 [113]. The occurrence of some potential bioactive compounds in this medicinal plant and their efficacy against SARS-CoV-2 must be tested for better drug therapy to manage COVID-19.

### 3.12. Alnus japonica *(Thunb.) Steud.*

*Alnus japonica* (*A. japonica*) belongs to *Betulaceae* family and originally it was found in Japan, Korea, China and Russia [128]. This medicinal plant has wide range of biological activities comprising antiviral, anticancer, anti-inflammatory, and antioxidant effects, as well as the induction of lymphatic and gastroenteric disorders [128,130]. It is mostly used for the treatment of various diseases such as fever, cancer, and blood, lymphatic and gastroenteric disorders [263]. Additionally, and most importantly, *A. japonica* has been known to have an antiviral effect on SARS-CoV, and its potent bioactive compounds include hirsutenone, oregonin, rubranoside rubranoside B, rubranol, and hirsutanonol, which are extracted from the bark of the *A. japonica* plant [130]. These bioactive components have also been recommended as a promising medicinal plant for the development of potential drugs against SARS-CoV PLpro. In 2012, Park et al. investigated the effect and efficacy of *A. japonica* bark against SARS-CoV. An in vitro study showed that *A. japonica* is able to inhibit the SARS-CoV PLpro with IC_50_ value ranging from 3 to 44.5 µM of these compounds (hirsutenone, oregonin, rubranoside rubranoside B, rubranol, and hirsutanonol) [130]. However, the mode of action suggested that *A. japonica* bark can possibly inhibit the SARS-CoV PLpro activity.

### 3.13. Psoralea corylifolia *L.*

*Psoralea corylifolia* (*P. corylifolia*) belongs to the *Leguminosae* family and mostly occurs in India, China, Bangladesh, Indonesia, Malaysia, Sri Lanka and other Asian countries. *P. corylifolia* is known for its antiviral, antioxidant, antibacterial and anti-depressant activities [132,133]. Furthermore, it contains several potent bioactive components such as neoba-vaisoflavone, isobavachalcone, Bavachinin, 40–O-methyl bavachalcone, corylifol A, and psoralidin [264]. In 2014, Kim et al. investigated the effect of *P. corylifolia* seed extract and showed an imperative inhibitory effect of SARS-CoV PLpro, and their IC_50_ was 15 µg/mL [134]. Furthermore, all these bioactive components were tested by Kim et al., and the IC_50_ of these components against SARS-CoV PLpro was estimated to range between 4.2 to 38.4 µM. In addition, psoralidin and isobavachalcone showed the highest inhibitory activity against SARS-CoV PLpro, with IC_50_ of 4.2 ± 1.0 µM and 7.3± 0.8 µM, respectively [134].

### 3.14. Paulownia tomentosa *(Thunb.) Steud.*

*Paulownia tomentosa* (*P. tomentosa*) is an old Chinese medicinal plant belonging to the *Scrophulariaceae* family. It is mostly found in central and western China, Taiwan and Korea. *P. tomentosa* has wide-ranging biological activities comprising antiviral, antioxidant and antibacterial effects [136,138]. It is mostly used for the treatment of various diseases, such as inflammatory bronchitis, upper respiratory tract infection, asthma, tonsillitis, gonorrhea, traumatic bleeding, enteritis, bacteriological diarrhea, erysipelas, swelling, bronchopneumonia, conjunctivitis, and hemorrhoids [139,265]. Furthermore, *P. tomentosa* has been known to have an antiviral effect on SARS-CoV PLpro. *P. tomentosa* fruit contains many bioactive components such as tomentin A, tomentin B, tomentin C, tomentin D, tomentin E, geranylated flavonones and others [137]. In 2013, Cho et al. examined the effect and efficacy of *P. tomentosa* fruit-extracted bioactive components against SARS-CoV. An in vitro study showed that *P. tomentosa* is able to inhibit SARS-CoV PLpro activity with an IC_50_ value ranging from 5.0 to 14.4 µM [137]. Out of all those studied, Tomentin E showed the most promising and highest inhibitory effect against SARS-CoV, with the lowest IC_50_ 5.0 ± 0.06 µM [137].

### 3.15. Tribulus terrestris *L.*

*Tribulus terrestris* (*T. terrestris*) is mostly found in China, India, Pakistan, South Americas, Bulgaria, Mexico and Spain, and is considered as a traditional medicinal plant. *T. terrestris* belongs to the *Zygophyllaceae* family and possesses several biological activities such as antiviral, anti-inflammatory, antioxidant, anti-tumor, anti-diabetic and anti-urolithic properties [141,143]. It contains several bioactive compounds, mainly flavonoids and alkaloids [142,266]. In 2014, Song et al. studied the effect of *T. terrestris* fruit extract (six cinnamic amides), and showed significant inhibitory effects against SARS-CoV PLpro [142]. Furthermore, all bioactive components tested by Song et al. against SARS-CoV PLpro were estimated to have an IC_50_ in a range between 15.8 and 70.1 µM [142]. However, terrestrimine[(E)-N-(1-hydroxy-2-(4-hydroxyphenyl)-2-oxoethyl)-3-(4-hydroxy3-methoxypheny) acrylamide] showed the utmost inhibitory activity against SARS-CoV PLpro with an IC_50_ of 15.8 ± 0.6 µM [142].

## 4. Other Medicinal Plants in Use against Various Viral Infections and Possibility for the Therapeutic Strategy against COVID-19

Currently, there are several plants which are clinically in use for the treatment of various diseases including viral infections. Considering their mode of action, potency and efficacy, here we have detailed some useful medicinal plants, which can possibly be used for the combinational therapeutic management of COVID-19 by inhibiting various protein targets of SARS-CoV-2.

### 4.1. Sambucus nigra *L.*

This plant belongs to the family of *Caprrifoliaceae* and is mostly used in the treatment of common cold, HIV, HSV-1, influenza, urinary tract infection, edema and other rheumatic diseases [149,267]. It contains several active components extracted from the leaves, flower and fruit parts of the plant, such as flavonoids, lectins, anthocyanin, etc., which have been found to increase the immunity and inhibit the viral activity [150,152,153,154]. However, in case of the H1N1 influenza virus, this plant has shown great potential to block or impede the entry of the virus into the host cells [149,155,268]. Furthermore, the presence of lectins in this plant is responsible for controlling the symptoms or pathogenesis of the influenza virus [149,151]. It also has immunomodulating activity due to the presence of peptic polysaccharides, polyphenolic compounds and flavonoids [149]. Due to several significant and antiviral relevant properties of this plant, it can possibly be used against SARS-CoV-2.

### 4.2. Eleutherococcus senticosus *(Rupr. & Maxim.) Maxim.*

*Eleutherococcus senticosus* (*E. senticosus*) belongs to the family of *Araliaceae* and is mostly found in China, Japan and Korea [269]. *E. senticosus* is used for the treatment of chronic coughing, ischemic heart disease, diabetes, cancer, altitude sickness, neurodegenerative disorders, and chronic fatigue [149,159,269,270]. Moreover, its leaves are used as food in the form of tea, wine, soups and many others [271]. *E. senticosus* leaves have the potential efficacy to inhibit bacterial glucosidase activity, reported in in vitro results by many researchers. The nature of the component of *E. senticosus* responsible for its antiviral activity remains to be determined, and is currently under investigation together with the characterization of the target molecules and the molecular basis of the antiviral efficacy of *E. senticosus*. However, its extract is able to inhibit the replication of the influenza virus, and viral replication is common in all kinds of viruses [157,158,159,272]. Several potent bioactive components are known to be present in the roots of this medicinal plant, such as phenols, lignans, coumarins, phenylpropanoids, flavonoids, hyperin, rutin, afzelin, quercetin, kaempferol, phenolic acids, triterpenic acids, and anthocyanin, etc. Due to the presence of these bioactive compounds, in vitro results showed some antiviral activity too, by blocking the replication of influenza virus in the cells [149,273]. Therefore, it is a possible recommendation that plant should go further investigation and may be helpful in directly or indirectly controlling SARS-CoV-2.

### 4.3. Salvia miltiorrhiza *Bunge*

This plant belongs to the family of *Lamiaceae* and is commonly known as red sage [274]. Its bioactive components are extracted from the root, including lipophilic diterpenoids, flavonoids, triterpenoids and hydrophilic phenolic compounds [163,274,275]. It is also used for the treatment of various diseases, such as removing blood stasis, improving blood circulation, atherosclerosis, thrombosis, angina pectoris, other cardiovascular diseases and antiviral activity of HIV-1 and Enterovirus by inhibiting RdRp enzyme activity [161,162,163,164].

### 4.4. Acacia arabica *(Lam.) Willd.*

*Acacia arabica* (*A. arabica*) belongs to the family of *Fabaceae* and is widely distributed in Asian regions [167]. It is basically used for the treatment of various diseases, such as Newcastle disease, vaccinia virus, bursal disease virus, H9N2 influenza disease, skin diseases, and possesses many biological properties including antimicrobial, anti-diabetic, and antioxidant effects. The mechanism of action of *A. arabica* is known. It specifically inhibits the stage of viral intracellular multiplication [149,167,168,169]. Furthermore, *A. arabica* contains several bioactive components extracted from the leaves of the plant which are responsible for its bioactivity, such as flavonoids, methyl 3,4,5 tri hydroxyl benzoate, *p*-coumaroyl glucoside, *p*-coumaroylquinic acid, ferulic acid, isoferulic acid, epicatechin-3-gallate, ascorbic acid, quercetin 3-O-(4′-O-acetyl)-rhamnopyranoside, oleic acid, myristic acid, palmitic acid and steroidal sapogenin aglycone [166,167,168,276]. *A. arabica* also has the potential to inhibit the viral replication against HIV infection [149,169]. Due to its antiviral nature, it is highly recommended to use *A. arabica* for controlling/managing SARS-CoV-2 infection.

### 4.5. Ocimum sanctum *L.*

*Ocimum sanctum* (*O. sanctum*) belongs to the family of *Lamiaceae* and is commonly known as tulsi [167,173]. This aromatic plant is basically found in all Asian countries. It is used for the treatment of diseases such as cough, anxiety, arthritis, dysentery, diarrhea, asthma, fever, skin and eye disorders, otalgia, gastrointestinal disorders, cardiac and genitourinary disorders, back pain, snake, insect and scorpion bites, malaria, and H9N2 influenza disease [16,167,168,173,178]. The leaves of *O. sanctum* contain several bioactive compounds, such as alkanoids, saponins, tannins, flavonoids, phenols, anthocyanins and triterpenoids [167,172,173]. However, this medicinal plant has the potential to block the activity of different pathogens and can act as a potent antiviral, antifungal, anti-protozoan, anti-malarial, anti-helminthic, antibacterial, mosquito repellent, etc.; its other clinical activities are detailed in Table 1 [167,171,172,173,174,175,176,177]. Ghoke et al. showed that treatment with the crude extract derived from the leaves of *O. sanctum* leads to significant H9N2 virus reduction in assessing all three—virucidal, therapeutic and prophylactic—activities using an in vivo model. They suggested that the crude extract of *O. sanctum* could be a promising extract for developing safe and efficacious antiviral compounds against the H9N2 virus. The protecting effectiveness of the crude extract of *O. sanctum* might be ascribed to multiple mechanisms of action (specific inhibition of viral intracellular multiplication stage and non-specific interference with virus–cell interactions such as masking/blocking the HA glycoprotein [167]. Due to these vast known biological properties, it would be of great importance to study the potential particular active ingredient or combinations, which are responsible for its broader antiviral activity, further. Therefore, *O. sanctum* might be helpful for the treatment of COVID-19, and can potentially block the entry of virus as well as its replication.

### 4.6. Ocimum basilicum *L.*

This medicinal herb belongs to the family of *Lamiaceae* and it is also known as sweet basil [149,181]. It is mostly used in industries as food, perfumes and cosmetics [181] due to its potent antiviral, anti-inflammatory, antioxidant and antibacterial activities [180,181,183,184]. Moreover, it contains several bioactive components such as phenolic compounds, flavonoids and anthocyanin extracted from the whole plant of *O. basilicum* [182,183,184]. This herb has been used for HIV treatment and showed very good potential to inhibit the replication of the HIV virus, and blocks further viral processing [149,181].

### 4.7. Theobroma cacao *L.*

*Theobroma cacao* (*T. cacao*) belongs to the family of *Sterculiaceae*. The seeds of this cocoa plant are commonly used in food industries [277]. It contains several types of bioactive compounds, such as polyphenol, theobromine and flavonoids [278], which are responsible for its antioxidant, antiviral, anti-inflammatory and many other biological activities [186,188,189]. However, some studies reported the anti-influenza activity of *T. cacao*, due to presence of flavonoids, theobromine, lignin, dietary fiber, free fatty acid, and minerals (zinc, copper, iron) [186,187,188]. Kamei et al., 2014 investigated the effect of *T. cacao* against the influenza virus and found that it enhances the antibody response due to stimulatory effect [187]. Furthermore, it has also been observed that, *T. cacao* helps in developing acquired immunity and activates the NK cells against the influenza virus [187]. Further investigation may lead to the use of *T. cacao* against SARS-CoV-2, and can help in boosting immunity.

### 4.8. Pelargonium sidoides *DC.*

*Pelargonium sidoides* (*P. sidoides*) belongs to the family of *Geraniaceae* and is commonly known as Umckaloabo [191]. It is found all over the world and roots of this plant are traditionally used for remedial purposes against tuberculosis, respiratory diseases, cough, gastrointestinal infection, viral diseases and others [191,193]. *P. sidoides* roots are known to have some potent compounds, such as methoxycoumarin, proanthocyanidins and prodelphinidins [192]. Furthermore, its roots are also used for the production of herbal drugs known as EPs 7639 by ethanolic extract, which have been approved for the treatment of respiratory tract infections [191,194]. According to Theisen et al., 2012, *P. sidoides* also has the potential to inhibit the viral entry of the influenza virus [194]. Therefore, it is suggested that roots of *P. sidoides* should be further investigated for the treatment of COVID-19.

### 4.9. Taraxacum officinale *(L.) WEB. ex WIGG.*

This medicinal plant belongs to the family of *Asteraceae* and it is commonly known as dandelion [48]. It is traditionally used for the treatment of various diseases such as kidney diseases, lung diseases, breast tumor, diabetes, uterus infections, digestive system related abnormalities, etc. [198,279]. Pharmacological research has proven the efficacy of this medicinal plant as antiviral, antibacterial, choleretic, anti-diabetic, anti-inflammatory, antioxidant, hepato-protective, diuretic and antifungal [198]. It contains several bioactive components, extracted from the aerial parts and roots, such as terpenes, flavonoids, phenolic compounds, terpenoids, triterpenoids, steroids, coumarins, phenols, saponins, flavones, flavonols, chalcones, phlobatannins, and cardiac glycosides [196,197,280]. Han et al., 2011 found the potential of this medicinal plant to inhibit the viral replication of HIV [199]. Similarly, Lee et al., 2012 also suggested its potential to enhance pro-inflammatory cytokines and improve the immune system [201]. Furthermore, it is also known to inhibit the influenza virus’ entry into cells [48,200]. Therefore, due to its vast and significant antiviral properties, it is highly recommended to conduct further investigation on this medicinal plant for the discovery of potent drugs against COVID-19.

### 4.10. Illicium oligandrum *Merr & Chun*

*Illicium oligandrum* (*I. oligandrum*) belongs to the family of *Magnoliaceae*, being a rich source of seco-prezizaane type sesquiterpenes [203]. It is known to have antiviral activity against herpes simplex virus type 2, coxsackie virus and influenza virus [204,206]. It has some potent bioactive compounds, such as sesquiterpene lactones, neolignan glycosides, phenolic diglycosides and prenylated compounds which are responsible for its antiviral activities [204,206]. However, this medicinal plant is also used for the treatment of rheumatoid arthritis, and neurotoxic and neuro-trophic effects [205]. Ma et al., 2013 reported the ethanolic extraction of spirooliganones A 1 & B from the roots of *I. oligandrum* and showed its potential to inhibit the activity of influenza virus (H3N2) (IC_50_ 3.70–33.33 µM) and coxsackie virus B3 [204].

### 4.11. Glycyrrhiza glabra *L.*

*Glycyrrhiza glabra* (Liquorice) belongs to the family of *Fabaceae* and is among the most ancient medicinal plants [212]. It has several very well-known biological activities, such as antiviral (HIV, SARS-CoV), anti-inflammatory, antimicrobial, antioxidant, anti-tumorigenic and anti-ulcer properties [209,210]. The root of Liquorice is known to have many bioactive components, including flavonoids, glycyrrhizic acid, triterpenoid, saponins, etc. [210,212]. Few studies showed that chalcones extracted from Liquorice have the ability to block or inhibit the activity of influenza virus [48,208]. Therefore, there is a possibility that this plant might be useful against SARS-CoV-2 due to its antiviral properties.

### 4.12. Angelica keiskei *(Miq.) Koidz.*

*Angelica keiskei* (*A. keiskei*) belongs to the family of *Umbelliferae,* and its leaves are basically used for remedial purposes [147]. Its bioactive components include chalcones, flavanones and coumarins, coumarins phenolic, acetylenes, sesquiterpene, diterpene, and triterpenes [144,146]. *A. keiskei* is known and considered to be antiviral, anticancer, anti-inflammatory, anti-obesity, anti-oxidative, anti-coagulant, anti-tumor, anti-mutagenic, anti-diabetic, antibacterial and hepato-protective [144,145,146]. Park et al., 2016 extracted bioactive components (9 alkylated chalcones and 4 coumarins) from *A. keiskei* plant [146], and revealed that the extracted chalcones were able to significantly block the entry of coronavirus (SARS-CoV-1) by inhibiting the chymotrypsin-like protease (75% inhibition using 30 µg/mL dose) and a papain-like protease (88% inhibition using 30 µg/mL dose) [146]. In addition, the IC_50_ of this chalcone and chalcone 6 are 11.4 and 1.2 µM, respectively [146]. Therefore, due to this very specific inhibition property of *A. keiskei* deserves further investigation for the development of potent antiviral agents against COVID-19.

### 4.13. Polygala karensium *Kurz*

*Polygala karensium* (*P. karensium*) is a medicinal plant belonging to the family of *Polygalaceae* and can mostly be found in China, Myanmar, Thailand, and Vietnam [215]. It has important and potent bioactive compounds, i.e., xanthones, which have shown many biological activities such as antiviral, antimicrobial, antioxidant, cytotoxicity, etc. [214,215,216]. In addition, it is also used to treat various ailments such as cough, bronchitis, neurasthenia, inflammation and amnesia [215]. However, Dao et al., 2012 conducted one in vitro study on H1N1, H9N2, and novel H1N1 (WT) strains using ethanol-extracted xanthones from the root of *P. karensium*, and found that xanthones have the potential to completely inhibit influenza virus activity [214]. Therefore, xanthones from *P. karensium* can also be one of the choices worth investigation for the further development of phytomedicine against SARS-CoV-2.

### 4.14. Calophyllum brasiliense *Cambess.*

*Calophyllum brasiliense* (*C. brasiliense*) is a medicinal plant, and basically belongs to the family of *Clusiaceae*, mostly found in South America, Central America and the Caribbean region [218]. It is used as a remedy to treat several diseases, such as parasitic, viral, bacterial and fungal diseases [218,220]. Its potent biological activities include antiviral, antibacterial, anti-protozoal and antifungal effects [218,219]. However, Kudo et al., 2013 investigated the role of this medicinal plant in HIV disease, firstly by extracting tricyclic coumarin from the leaves of *C. brasiliense* and testing in vitro [220]. Hence, they revealed that tricyclic coumarin from *C. Brasiliense* possesses great potential to inhibit viral replication by blocking the NFkB pathway [220].

### 4.15. Cimicifuga foetida *L.*

*Cimicifuga foetida* (*C. foetida*) belongs to the family of *Ranunculaceae* and it is also known as Shengma. It is abundantly distributed in Asian region [225]. It is basically used to treat various ailments, such as fever, headache, sore throat, toothache, uterine prolapse and inflammation [224]. It contains several bioactive compounds extracted from rhizomes, including cycloartane triterpenoids and glycosides with antiviral, anti-tumor, anti-inflammatory activities [225]. Wang et al., 2012 investigated the role of *C. foetida*, especially the cimicifugin component of it, against Respiratory Syncytial Virus, and found that the plant has a strong potential to inhibit viral attachment and internalization [223,224]. Moreover, cimicifugin was also able to stimulate epithelial cells and initiate the secretion of cytokines such as IFN-β, to clear the viral infection/load [281]. Furthermore, another in vitro study conducted by Dai et al., 2016 observed the potential of *C. foetida* in inhibiting the hepatitis B virus transcription and replication by producing pro-inflammatory cytokines [222]. Due to the capacity of producing strong pro-inflammatory cytokines and immunomodulatory properties, *C. foetida* can be used to treat COVID-19 disease.

### 4.16. Boerhavia diffusa *L.*

*Boerhavia diffusa* (*B. diffusa*) belongs to the family of *Nyctaginaceae* and is commonly known as punarnava [282]. It is mostly found in Asian countries and is basically used for the treatment of various diseases, such as abdominal pain, jaundice, dyspepsia, stress, spleen enlargement and liver diseases [228]. *B. diffusa* bioactive components extracted from leaf, stem and root include flavonoids, triterpenoids, alkaloids, hypoxanthine, steroids, lipids, lignin, proteins, ursolic acid, boeravinone, punarnavoside, etc. [227,230]. However, Bose et al., 2017 suggested that *B. diffusa* has a strong potential to inhibit the entry of hepatitis C virus and its major compound (boeravinone H component) were able to block the initial phase of HCV entry through acting directly on the viral particles [228]. Moreover, Manu et al., 2007 also showed that its second major bioactive compound (Punarnavine) was also able to enhance the immune response, especially IFN-γ and interleukin-2 cytokines [229]. This categorizes *B. diffusa as* a therapeutically important plant to be considered under the current circumstances of the COVID-19 pandemic and worth further investigation.

### 4.17. Terminalia chebula *Retz*

*Terminalia chebula* (*T. chebula*) belongs to the family of *Combretaceae* and is mostly found in the Asian region [283]. It is one of the most important medicinal plants due to the presence of a huge number of different kinds of phytoconstituents [232]. It is customarily used as a household remedy and also in modern, Ayurveda, Unani and Homoeopathic medicines [232,283]. Its bioactive components extracted from the leaves, bark and fruit of the plant include flavonoids, polyphenols, terpenes, anthocyanins, glycosides, gallic acid, chebulagic acid, punicalagin, chebulanin, corilagin, neochebulinic acid, ellagic acid, chebulinic acid, alkaloids and many more [232,237,284]. It is also known to be used as a cure for irregular fevers, urinary diseases, diabetes, skin diseases, heart diseases, constipation, ulcers, vomiting, colic pain, hemorrhoids, digestive diseases, and others [232,235,237]. However, *T. chebula* has many pharmacological activities such as antiviral, antioxidant, antibacterial, antifungal, anti-protozoal, anti-carcinogenic, anti-mutagenic, radio-protective, chemo-preventive, hepato-protective, cardio-protective, cyto-protective, anti-diabetic, reno-protective, anti-inflammatory, anti-arthritic, adaptogenic, anti-anaphylactic, hypolipidemic, hypocholesterolemic, anti-caries, wound healing, anti-allergic, immunomodulatory, anti-ulcer, anti-spasmodic and gastrointestinal motility properties [232,233,235,236,237]. Lin et al., 2013 conducted an in vitro study and found that chebulagic acid and punicalagin from the fruit of *T. chebula* have the potential to inhibit the activity of different viruses, such as human cytomegalovirus, HCV, dengue virus, measles virus, and respiratory syncytial virus [234]. Due to its strong biological properties, it is highly recommended to study *T. chebula* as a possible remedy against SARS-CoV-2.

### 4.18. Caesalpinia sappan *L.*

*Caesalpinia sappan* (*C. sappan*) belongs to the family of *Caesalpiniaceae* and it is usually known as Brazil or Sappan wood [219]. It is mostly found in Southeast Asian regions and is traditionally used for the treatment of various diseases such as tuberculosis, diarrhea, dysentery, skin infections, anemia, etc. [219,240]. *C. sappan* is effectively considered as an antiviral, anti-inflammatory, antioxidant, antibacterial, antifungal, and anti-complementary [219,240,241]. Its bioactive constituents include xanthone, coumarin, chalcones, flavones, homoisoflavonoids, and brazilin [240]. Tewtrakul et al., 2015, extracted nine compounds from the roots of *C. sappan*. The results showed that, out of those nine, sappanchalcone (IC_50_ 2.3 µM) and protosappanin A (IC_50_ 12.6 µM) presented the strongest effect against HIV-1 IN [240]. On the other hand, Liu et al., 2009 also investigated the role of this medicinal plant against influenza virus. The in vitro study showed that 3-deoxysappanchalcone and sappanchalcone component isolated from *C. sappan* exhibited the highest activity against influenza virus (H3N2), with IC_50_ 1.06 and 2.06 μg/mL, respectively [239]. Therefore, sappanchalcone from *C. sappan* should also be considered for further examination against SARS-CoV-2.

## 5. Plant-Based Antiviral Drug Discoveries and Future Perspectives

There are several plant-based natural compounds which are either under investigation, in preclinical trials or in clinical trials. Two such plant-based antiviral compounds under clinical trials are (+)-Calanolide A and SP-303. Calanolide A is isolated from the *Calophyllum langigerum,* and is mostly found in Malaysia. It is a C22 coumarin mostly used for the treatment of HIV infection [285,286,287]. This natural product has completed the phase 1 clinical trial (NCT00002243). On the other hand, SP-303 is isolated from the latex of a Latin American plant *Croton lechleri.* However, this natural compound is the mixture of oligomeric proanthocyanidins with a molecular weight of 2100 daltons [285,288]. SP-303 is used for the treatment of HSV and HIV infection. It is currently under clinical trial (NCT00002408). Furthermore, seven known FDA-approved antiviral drugs (famciclovir, ganciclovir, sorivudine, zidovudine, didanosine, zalcitabine and stavudine) are originally modeled on a natural product parent [285].

It is strongly believed that natural compounds or drugs have good potential to effectively treat virus-related diseases. Though the process of drug development from the natural product is time-consuming, their efficacy is known to produce long-term effectiveness. Recent developments in advance instrumentations and novel techniques helped in identifying various novel and effective plant-based natural drugs, leading to the development of potent antiviral compounds. Moreover, they can also be referred as supplementary foods or nutraceuticals due to their therapeutic potential, which can ultimately aid in promoting good health. Therefore, in vivo model studies, safety certifications, and mandatory clinical trial implementations of natural compounds should fulfill the emergency demands against serious diseases. This will also directly or indirectly contribute to controlling the pandemic situation.

## 6. Conclusions

Medicinal plants and their natural bioactive compounds provide significant and powerful resources, displaying diverse antiviral properties. Their biodiversity serves as an excellent source of new antiviral drugs, revealing new chemical structures that can act on various biochemical pathways, leading to the development of novel and effective therapeutic drugs against viral diseases. Some medicinal plants and their natural products are identified to possess robust antiviral activities, mainly against coronaviruses, HIV and influenza. It is necessary that their identification/discovery should go for further investigations to provide the population with therapeutic agents against SARS-CoV-2 with increased efficiency and compliance. Most of the studies in this field are still in the initial stages of research, with a few being in in vivo experiments and clinical trials. Therefore, it is very important to not ignore the potency of medicinal plants that should be further investigated and explored in characterizing specific bioactive agents, as well as their mechanism, efficacy, and application through in vivo studies. This will lead us to natural therapeutic approaches against various infectious diseases including COVID-19. In addition, these plants can also be used in combinational therapy, due to the presence of natural compounds which can act as immunomodulators and might be helpful in combatting the diseases in a natural way, or can work as adjuvants to create a good drug therapy. We strongly believe that phytomedicines will play an imperative role and continue to support in developing potential drugs against SARS-CoV-2.

## Figures and Tables

**Table 1:** List of medicinal plants used for the treatment of MERS-CoV, SARS-CoV-1 and other viral infections with their potent bioactive compounds, biological activities and therapeutic effect against various diseases. These plants can possibly be used to target SARS-CoV-2.

Botanical Name and Vegetal Part Use for Medicinal Purpose	Picture	Bioactive Compounds	Biological Activities	Therapeutic Effect against Diseases	References
**Medicinal Plants Against Coronavirus Related Infections**					
*Bupleurum* species (Root)	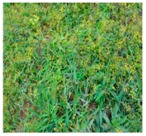	SSa, SSb2, SSc and SSd	Antiviral, anti-inflammatory, anti-tumor, neuro-modulation, immune-regulation	CoVs and Influenza virus	[32,34,35]
*Lycoris radiate* (L’Hér.) Herb. (Flower and stem cortex)	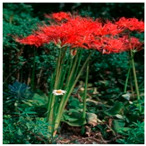	Lycorine	Antiviral effects, anticancer, anti-malarial, anti-inflammatory, induction of nausea and emesis	SARS-CoV-1, poliovirus, HIV, HSV and coxsackie virus	[41,42,43,44,71]
*Artemisia annua* L. (Whole plant)	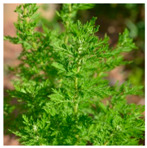	Quercetin, flavonoid, polyphenols, triterpenes, sterols, saponins, polysaccharides, dicaffeoylquinic acid	Anti-malarial, antiviral, anticancer, bronchitis, haemorrhoids	SARS-CoV-1, MERS-CoV, Poliovirus, HIV, RSV, HSV1, hepatitis C, type 2 dengue virus and human cytomegalovirus	[29,45,48,49,50,72]
*Pyrrosia lingua*(Thunb.) Farw. (Leaves)	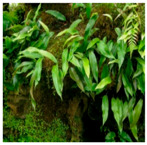	Flavonoids, (mangiferin, isomangiferin, trifolin, astragalin), chlorogenic acid, mangiferin, isomangiferin, astragalin, and trifolin	Antiviral, antioxidant, antibacterial, anticancer	HIV, SARS-CoV-1	[48,50,52,54,73]
*Isatis indigotica* Fortune ex Lindl. (Leaf and root)	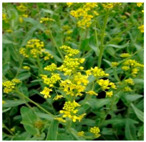	Indigo, indirubin, indican, β-sitosterol, sinigrin, hesperetin, aloe-emodin	Antiviral, antioxidant, antibacterial	SARS-CoV-1 3CLpro, HSV1, Influenza virus, coxsackie virus B3	[48,55,57,58,74]
*Torreya nucifera* L. (Leaves)	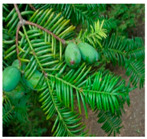	Biflavonoid amentoflavone	Antiviral	SARS-CoV-1 3CLpro, stomachache, hemorrhoids, and rheumatoid arthritis	[48,59,61,75]
*Houttuynia cordata* Thunb. (Leaves)	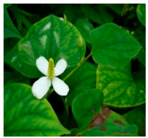	Volatile oils, organic acids, flavonoids cordarine, kalium sulfuricum, potassium, zinc, iron, copper, amino acid, vitamins and manganese	Antiviral, anti-inflammatory, anti-allergic, anti-oxidant, Immunomodulatory and anticancer	SARS-CoV-1 3CLpro and RdRp, cough, lung abscess, phlegm, dyspnea, pneumonia, refractory hemoptysis	[48,57,63,65,66,76]
*Lindera aggregate* (Sims) Kosterm. (Root)	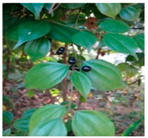	Flavonoids, isoquinoline alkaloids, sesquiterpene lactones and tannins	Antiviral, anti-tumor, anti-inflammatory, antimicrobial and anti-diabetic	SARS-CoV-1, chest pain, inflammation, indigestion, cold hernia	[48,50,68,69,77]
*Rheum palmatum* L. (Root)	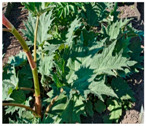	Emodin, physcion, chrysophanol, rhein, and aloe-emodin	Antiviral, anti-pyretic, anti-neoplastic, anti-pasmolytic, antibacterial, laxative, hemostatic, and anti-spasmodic	SARS-CoV-1 ACE2, laxative or astringent, stomachicum, hemorrhoids, liver bile disease or gastroenteritis	[78,79,80,81,82,83]
*Polygonum multiflorum* Thunb. (Root)	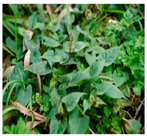	Polygonumosides A, B, C, and D, resveratrol, chrysophanol, polydatin, emodin-1,6-dimethyl ether, rhaponticoside, emodin, 2-acetylemodin, physcion, rhein, citreorosein, apigenin, fallacinol, tricin, rutin, quercetin, luteolin, kaempferol, iso-orientin, hyperoside, vitexin, quercetin-3-O-arabinoside, polygonflavanol A, hexadecanoic acid ethyl ester, phosphatidylethanolamine, hexanoic acid, copaene, eicosane, squalene, catechin, epicatechin, 3-O-galloyl-procyanidin B2, *β*-sitosterol, gallic acid, methyl gallate, daucosterol, and schizandrin	Anti-CoVs, antioxidant, immunomodulation, anti-hyperlipidemia, anticancer, hepato-protection, anti-inflammation,	SARS-CoV-1 ACE2, rubella, scrofula, waist and knee pain, paralysis, vaginal discharge, hypercholesterolemia (liver and kidney), malaria, neuro-protective	[78,84,85,86,87,88,89]
*Cerasus avium* (L.) Moench (Stem)	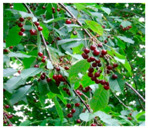	Polyphenols, carotenoids, vitamins, minerals	Antioxidant, antimicrobial and antiviral	SARS-CoV-1 ACE2, oxidative stress, tooth aches and mouth diseases	[78,90,91,92,93,94,95]
*Alcea digitata* (Boiss.) Alef (Flower)	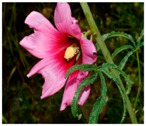	Unknown	Antiviral, antioxidant, anti-inflammatory, antimicrobial, anti-tussive, expectorant and laxative	SARS-CoV-1 ACE2, lung respiratory disorder, head and neck cancer and lubrication of throat	[91,92,96,97,98]
*Citrus aurantium* L. (Fruit)	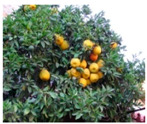	Phenolics (flavanone glycosides, hydroxycinnamic acids), vitamin C, and carotenoids	Antiviral, antioxidant, anticancer	SARS-CoV-1 ACE2, anxiety, lung related disease, obesity, gastrointestinal disorder and prostate cancer	[91,92,99,100,101,102]
*Rubia tinctorum* L. (Root)	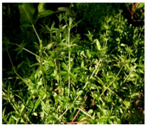	Anthraquinone, Alizarin and pseudopurpurin	Antiviral, antimicrobial	SARS-CoV-1 ACE2, kidney, bladder stone, menstrual and urinary disorder	[91,92,103,104,105]
*Allium sativum* L. (Cloves)	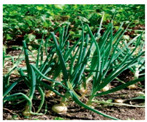	Alliin, allicin, ajoenes, vinyldithiins, and flavonoids	Antiviral, antimicrobial, antioxidant, anti-inflammatory, and anticancer	SARS-CoV-1 ACE2, inflammation, cancer and bacterial infection	[91,92,94,106,107,108,109,110]
*Quercus infectoria* G. Olivier (Gall)	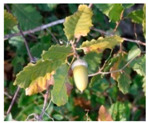	Phenolic compound (*p*-hydroxybenzoic acid, catechol, caffeine, pyrogallol, catechein, e-vanillic acid, 3-hydroxytyrosol cinnamic, *p*-Coumaric, gallic acids and resveratrol), flavonoid compounds	Antiviral, anti-fungal, antibacterial, antioxidant, anti-inflammatory, anti-diabetic, anti-parasitic, anti-venom	SARS-CoV-1 ACE2, diarrhea, menorrhagia, dysentery, gonorrhea, tonsillitis, impetigo and internal hemorrhages	[111,112,113,114,115]
*Onopordum acanthium* L. (Leaf, flower, stem and root)	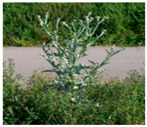	Flavonoids, sesquiterpene lactones, lignans, phenylpropanoids, triterpenoids, and sterols	Antiviral, anti-tumor, anti-inflammatory, antioxidant and cardio-tonic agent	SARS-CoV-1 ACE2, cancer, treat nervousness	[113,116,117,118,119]
*Berberis integerrim* Bunge (Root)	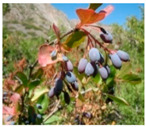	Berbamine, berberuin, palmatine, oxyacanthine, malic acid, ascorbic acid, caffeic acid, ursolic acid, coumarin, beta carotene, and tannin	Antiviral, anti-inflammatory, anti-hyperglycemic, anti-hyperlipidemic, anticancer, antioxidant	SARS-CoV-1 ACE2, alleviate insomnia, bronchial diseases, and liver disorder	[113,120,121,122,123]
*Crataegus microphylla* C. Koch (Leaves, flower, stem and root)	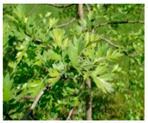	Phenols, phenolic acids, procyanidins, flavonoids, triterpenes, polysaccharides, catecho-lamines	Antiviral, antioxidant, anti-inflammatory and anti-diabetic	SARS-CoV-1 ACE2, heart muscle cells activation, coronary dilation, regulated blood flow	[113,120,124,125,126,127]
*Alnus japonica* (Thunb.) Steud. (Bark)	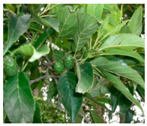	Hirsutenone, oregonin, rubranoside rubranoside B, rubranol, and hirsutanonol	Antiviral, anticancer, anti-inflammatory, antioxidant and induction of lymphatic and gastroenteric disorders.	SARS-CoV-PLpro fever, cancer, blood and lymphatic disorders, gastroenteric disorders	[128,129,130]
*Psoralea* *corylifolia* L. (Seed)	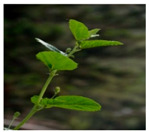	Neobavaisoflavone, isobavachalcone, Bavachinin, 40 –O-methyl bavachalcone, corylifol A and psoralidin	Antiviral, antioxidant, antibacterial and anti-depressant activities	SARS-CoV-PLpro leukoderma, psoriasis, vitiligo, asthma, ulcers, kidney disorders	[131,132,133,134]
*Paulownia tomentosa* (Thunb.) Steud. (Fruit)	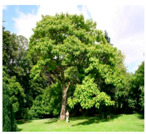	Tomentin A, tomentin B, tomentin C, tomentin D, tomentin E, geranylated flavonones	Antiviral, antioxidant and antibacterial	SARS-CoV-PLpro inflammatory bronchitis, upper respiratory tract infection, asthma, tonsillitis, gonorrhea, traumatic bleeding, enteritis, bacteriological diarrhea, erysipelas, swelling, bronchopneumonia, conjunctivitis, and hemorrhoid	[135,136,137,138,139]
*Tribulus terrestris* L. (Fruit)	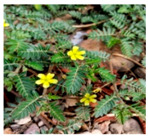	Flavonoid and alkaloids	Antiviral, anti-inflammatory, antioxidant, anti-tumor, anti-diabetic and anti-urolithic	SARS-CoV-PLpro hypertension, premature ejaculation, erectile dysfunction, vitiligo, and kidney	[140,141,142,143]
*Angelica keiskei* (Miq.) Koidz. (Leaves)	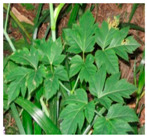	Chalcones, flavanones and coumarins, coumarins phenolic, acetylenes, sesquiterpene, diterpene, and triterpenes	Antiviral, antibacterial, anti-diabetic, anticancer, anti-inflammatory, antioxidative, anti-coagulant, anti-obesity, anti-tumor, anti-mutagenic and hepato-protective	SARS-CoV-1 3CLpro, bacterial treatment, cancer and diabetes	[144,145,146,147,148]
**Medicinal Plants against Other Viral Infections**					
*Sambucus nigra* L. (Leaf, flower and fruit)	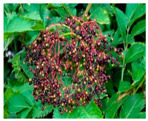	Flavonoids, lectins, anthocyanin, peptic polysaccharides, polyphenolic compound	Antiviral, Immunomodulatory activity, anti-inflammatory	Common cold, HIV, HSV1, influenza, urinary tract infection, edema, rheumatic	[149,150,151,152,153,154,155,156]
*Eleutherococcus senticosus* (Rupr. & Maxim.) Maxim. (Leaf and root)	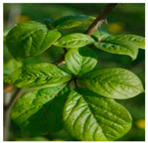	Phenols, lignans, coumarins, phenylpropanoids, flavonoids, hyperin, rutin, afzelin, quercetin, kaempferol, phenolic acids, triterpenic acids, and anthocyanin	Antiviral, anti-diabetic, anticancer, antioxidant	Influenza virus, chronic coughing, fatigue and infection, ischemic heart disease, diabetic, cancer, altitude sickness, neurodegenerative disorder	[101,149,157,158,159,160]
*Salvia miltiorrhiza* Bunge (Root)	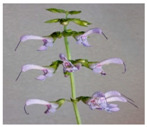	Lipophilic diterpenoids, flavonoids, triterpenoids and hydrophilic phenolic compound	Antiviral	HIV, enterovirus removing blood stasis, improving blood circulation, atherosclerosis, thrombosis, angina pectoris, cardiovascular disease	[161,162,163,164,165]
*Acacia arabica* (Lam.) Willd (Leaves)	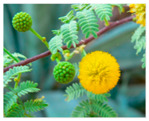	Methyl 3,4,5 tri hydroxyl benzoate, ferulic acid, p -coumaroylquinic acid, isoferulic acid, *p*-coumaroyl glucoside, epicatechin-3-gallate, ascorbic acid, quercetin, oleic acid, myristic acid, palmitic acid and steroidal sapogenin aglycone	Antiviral, antimicrobial, anti-diabetic, antioxidant	HIV, influenza virus, Newcastle disease, vaccinia virus, bursal disease virus, skin disease	[149,166,167,168,169,170]
*Ocimum sanctum* L. (Leaves)	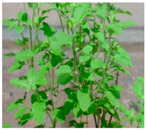	Flavonoids, tannins, saponins, alkaloids, phenols, anthocyanine, triterpenoids,	Antiviral, antimicrobial, anti-cataract, anti-inflammatory, anti-diabetic, anti-hypercholesterolemia, anti-hypertensive, anti-carcinogenic, anti-pyretic, anti-allergic, immunomodulatory, anti-asthmatic, anti-tussive, anti-fertility, anti-ulcer, anti-emetic, anti-spasmodic, anti-arthritic, adaptogenic, anti-leukodermal, anti-coagulant activities	H9N2 influenza disease anxiety, cough, asthma, diarrhea, fever, skin disease, dysentery, arthritis, eye diseases, otalgia, indigestion, hiccups, vomiting, gastric, cardiac and genitourinary disorders, back pain, skin diseases, ringworm, insect, snake and scorpion bites, malaria and antioxidant	[167,171,172,173,174,175,176,177,178,179]
*Ocimum basilicum* L. (Whole plant)	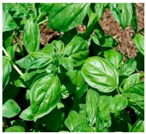	Phenolic compounds, flavonoids and anthocyanins	Antiviral, anti-inflammatory, antioxidant and antibacterial	HIV infection and bacterial infection	[149,180,181,182,183,184,185]
*Theobroma cacao* L. (Seed)	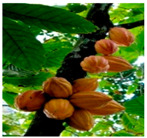	Polyphenol, theobromine and flavonoids (heobromine, lignin, dietary fiber, free fatty acid, minerals, zinc, copper, iron)	Antiviral, antioxidant, anti-inflammatory	Influenza virus	[186,187,188,189,190]
*Pelargonium sidoides* DC (Root)	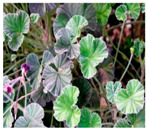	Methoxycoumarin, proanthocyanidins, EPs 7639 and prodelphinidins	Antiviral and antioxidant	Influenza virus, tuberculosis, respiratory disease, cough, gastrointestinal infection, viral disease	[191,192,193,194,195]
*Taraxacum officinale* (L.) WEB. ex WIGG. (Aerial part and root)	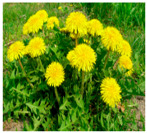	Terpenes, flavonoids, phenolic compounds, terpenoids, triterpenoids, steroids, coumarins, phenols, saponins, flavones, flavonols, chalcones, phlobatannins, and cardiac glycosides	Antiviral, antibacterial, choleretic, anti-diabetic, anti-inflammatory, antioxidant, hepato-protective, diuretic and antifungal	HIV, influenza virus, kidney related disease, lung related disease, tumor of breast, diabetic, uterus related infection, digestive system related abnormality	[48,196,197,198,199,200,201,202]
*Illicium oligandrum* Merr and Chun (Root)	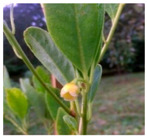	Sesquiterpene lactones, neolignan glycosides, phenolic diglycosides and prenylated compounds	Antiviral	HSV, coxsackie virus and influenza virus, rheumatoid arthritis, neurotoxic and neurotrophic effects.	[203,204,205,206,207]
*Glycyrrhiza glabra* L. (Root)	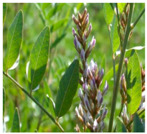	Flavonoids, glycyrrhizic acid, triterpenoid, saponins	Antiviral, anti-inflammatory, antimicrobial, antioxidant, anti-tumorigenic and anti-ulcer	CoVs, HIV, influenza virus	[208,209,210,211,212,213]
*Polygala karensium* Kurz (Root)	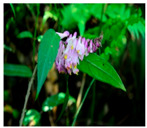	Xanthones	Antiviral, antimicrobial, antioxidant, cytotoxicity activity	Influenza virus, cough, bronchitis, neurasthenia, inflammation and amnesia	[214,215,216,217]
*Calophyllum brasiliense* Cambess (Leaves)	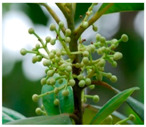	Tricyclic coumarin	Antiviral, antibacterial, anti-protozoal and antifungal	HIV, parasitic diseases, bacterial and fungal disease	[218,219,220,221]
*Cimicifuga foetida* L. (Rhizomes)	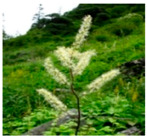	Cimicifugin, cycloartane triterpenoids and glycosides	Antiviral, anti-tumor, anti-inflammatory	Respiratory Syncytial Virus, fever, headache, sore throat, toothache, uterine prolapse and inflammation	[222,223,224,225,226]
*Boerhavia diffusa* L. (Leaf, stem and root)	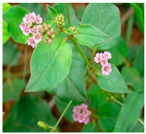	Flavonoids, triterpenoids, alkaloids, hypoxanthine, steroids, lipids, lignins, ursolic acid, boeravinone, punarnavoside	Antiviral, anti-fibrinolytic, anti-convulsant, antibacterial, anti-hepatotoxic, anti-asthmatic and anti-nematodal activity	Hepatitis C virus, abdominal pain, jaundice, dyspepsia, release the stress, spleen enlargement, liver	[227,228,229,230,231]
*Terminalia chebula* Retz (Leaf, bark and fruit)	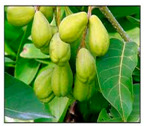	Flavonoids, polyphenols, terpenes, anthocyanins, glycosides, gallic acid, chebulagic acid, punicalagin, chebulanin, corilagin, neochebulinic acid, ellagic acid, chebulinic acid, alkaloids	Antiviral, antioxidant, antibacterial, antifungal, anti-protozoal, anti-carcinogenic, anti-mutagenic, anti-diabetic, reno-protective, anti-inflammatory, anti-arthritic, anti-anaphylactic, anti-caries, anti-allergic, immunomodulatory, anti-ulcer, anti-spasmodic	Human cytomegalovirus, hepatitis C virus, dengue virus, measles virus, respiratory syncytial virus, irregular fevers, urinary diseases, diabetes, skin diseases, heart diseases, constipation, ulcers, vomiting, colic pain, hemorrhoids, digestive diseases	[232,233,234,235,236,237,238]
*Caesalpinia sappan* L. (Root)	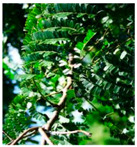	Xanthone, sappanchalcone, coumarin, chalcones, flavones, homoisoflavonoids, and brazilin	Antiviral, anti-inflammatory, antioxidant, antibacterial, antifungal, anti-complementary	HIV, Influenza virus, tuberculosis, diarrhea, dysentery, skin infections and anemia	[219,239,240,241,242]
